# Prospective associations of multidimensional well-being with work distraction and job satisfaction: a two-wave study of US employees

**DOI:** 10.3389/fpsyg.2024.1326655

**Published:** 2024-01-26

**Authors:** Eric N. Fung, Richard G. Cowden, Ying Chen, Dorota Weziak-Bialowolska, Piotr Bialowolski, Matthew T. Lee, Eileen McNeely, Tyler J. VanderWeele

**Affiliations:** ^1^Department of Epidemiology, Harvard T.H. Chan School of Public Health, Boston, MA, United States; ^2^Human Flourishing Program, Institute for Quantitative Social Science, Harvard University, Cambridge, MA, United States; ^3^Sustainability and Health Initiative (SHINE), Department of Environmental Health, Harvard T.H. Chan School of Public Health, Boston, MA, United States; ^4^Department of Quantitative Methods and Information Technology, Kozminski University, Warsaw, Poland; ^5^Department of Economics, Kozminski University, Warsaw, Poland; ^6^Institute for Studies of Religion, Baylor University, Waco, TX, United States

**Keywords:** human flourishing, well-being, social connectedness, emotional health, work distraction, job satisfaction, longitudinal

## Abstract

Previous studies on the associations between well-being and work outcomes, such as work distraction and job satisfaction, have largely been cross-sectional and typically focused on only one or two aspects of well-being. Using two waves of data from a sample of employees at a United States health insurance company (*n* = 1,234), the present brief research report examines prospective associations between six domains of well-being (emotional health, physical health, meaning & purpose, character strengths, social connectedness, and financial security) and two work outcomes (work distraction and job satisfaction). Lagged regression analyses provided some evidence indicating that higher-level well-being in several domains was associated with subsequent reduced work distraction and increased job satisfaction assessed approximately 1 year later, but the magnitude of associations with each outcome did vary by specific domain. Emotional health and social connectedness were most strongly associated with work distraction and job satisfaction. We discuss some implications of the findings, including the importance of applying a multidimensional approach to studying employee well-being and potential opportunities for organizations to support the well-being of their employees.

## 1 Introduction

Well-being is a multidimensional concept that refers to “the quality of one’s personal subjective state across the physical, mental, social, and spiritual dimensions of existence” (Lomas and VanderWeele, 2022, p. 5). Although the notion of “complete well-being” is a desirable end in its own right (Lee et al., 2021, 2022), well-being itself may have important consequences for different aspects of a person’s life, such as work (Schulte et al., 2015; Litchfield et al., 2016; Weziak-Bialowolska et al., 2020b). In this brief report, we use two waves of observational data to examine the associations between multidimensional well-being and work-related outcomes in a sample of United States employees.

Previous studies have addressed the implications of well-being for work-related functioning. For example, a longitudinal study showed that happiness and life satisfaction increased subsequent job happiness and job satisfaction, that depression in life increased subsequent depression at work, and that meaning in life increased subsequent meaning at work (Weziak-Bialowolska et al., 2020b). Other research suggests that well-being may have both proximal (individual level) and distal (organizational level) effects on work outcomes. Cross-sectional studies suggest that better physical health (Boles et al., 2004), emotional well-being (Russell, 2008), and psychological well-being (Wright and Cropanzano, 2000) are positively related to work outcomes, such as productivity, performance, and job satisfaction. More recently, a longitudinal study found that both physical health and mental health problems predicted subsequent decreased worker productivity (Bryan et al., 2022), which in turn may lead to higher indirect costs for organizations (Hemp, 2004; Lohaus and Habermann, 2019). Indeed, employee distraction can result in financial loss for the organization, to an even greater extent than financial loss associated with absenteeism (Gill et al., 2012; Bialowolski et al., 2020). Thus, existing evidence indicates that worker well-being matters for the individual employee, as well as for the organization overall. Furthermore, another longitudinal study found that a psychological climate for caring at work at baseline increased subsequent self-reported work productivity and quality, including less distraction (Weziak-Bialowolska et al., 2020a, 2023), suggesting that organizations can promote emotional health to benefit both their employees and their own productivity. Although prior research suggests that well-being may have a small to moderate effect on both job satisfaction (Bowling et al., 2010) and work performance (Tenney et al., 2016), more information about the specific aspects of well-being that affect work outcomes is needed. In order to optimize support for their employees and improve work outcomes, organizations need clear, consistent, and compelling evidence about the implications of employee well-being for work performance.

The present brief research report uses a two-wave prospective cohort of employees to examine the associations between domains of well-being and two commonly used indicators of employee work performance, namely work distraction and job satisfaction. In doing so, this research addresses at least three gaps in the existing literature on well-being and work outcomes. First, whereas prior studies often employed cross-sectional designs, the current study uses a longitudinal approach, which provides stronger causal evidence. Second, previous studies in this area have focused primarily on a single or few indicators of well-being as predictors of work outcomes (primarily mental health), whereas our multidimensional approach to measuring well-being helps paint a more holistic picture of how well-being shapes work outcomes. It is useful to explore both a multidimensional conceptualization of well-being and the measurement of multiple domains of well-being because different domains of well-being could vary in their implications for work performance and satisfaction. Third, to our knowledge, relatively few longitudinal studies consider well-being as a predictor rather than an outcome. Although well-being is an important end in and of itself, well-being may also shape work outcomes (Russell, 2008; Bowling et al., 2010; Tenney et al., 2016; Weziak-Bialowolska et al., 2020b; Bialowolski and Weziak-Bialowolska, 2021). Whereas some studies have broached certain aspects of well-being (e.g., psychological) as a predictor of work outcomes, research with a systematic measure of multidimensional well-being is more limited. Based on previous studies, we expected to find evidence for an association between well-being at baseline and a decrease in work distraction and an increase in job satisfaction at follow-up. However, prior literature suggests that the strength of these associations might vary by domain of well-being (e.g., Charalampous et al., 2019); hence, we anticipated that some domains of well-being might evidence stronger associations with work outcomes than other domains.

## 2 Methods

### 2.1 Study sample

The present brief research report leveraged two waves of data from a random sample of employees working at a large, 15,000-employee, national, self-insured services organization in the United States. All employees received an email invitation to participate in the survey, to which 2,364 participants provided baseline data (T1: June 2018, T2: July 2019). At T1, the sample was reasonably representative of the organization’s total workforce (Lee et al., 2021; Chen et al., 2023). Of the participants who responded at T1, those who participated at T2 (*n* = 1,411; 59.7% retention) were more likely to be older, female, non-Hispanic White, married, and a homeowner compared to participants who dropped out (see Chen et al., 2022). There was little evidence of differences between the retained and non-retained participants on other major sociodemographic characteristics. The analytic sample in this study included participants who responded to both T1 and T2 surveys and had complete data on the outcome variables (*n* = 1,234). Missing data on the predictor and covariates were imputed by multiple imputation. This study was approved by the Harvard T.H. Chan School of Public Health Institutional Review Board. Written informed consent was obtained from all participants.

### 2.2 Predictors (T1)

#### 2.2.1 Domains of well-being

Participants completed the previously validated 40-item Well-Being Assessment (WBA; Lee et al., 2021; Weziak-Bialowolska et al., 2021; Chen et al., 2022). The instrument assesses well-being in six domains: emotional health (7 items; e.g., “How satisfied are you with life as a whole these days?”), physical health (7 items; e.g., “How would you rate your physical health?”), meaning & purpose (6 items; e.g., “I have values and beliefs that help me understand who I am”), character strengths (7 items; e.g., “I always act to promote good in all circumstances, even in difficult and challenging situations”), social connectedness (7 items; e.g., “My relationships are as satisfying as I would want them to be”), and financial security (6 items; e.g., “I am able to meet my normal monthly living expenses without any difficulty”). Factor analytic evidence with this sample provided support for the six-factor structure of the WBA (Weziak-Bialowolska et al., 2021). Each item is rated using an 11-point response scale from 0 to 10 (see Supplementary Table S1). Higher scores imply greater well-being. We averaged responses to each of the domains to calculate domain-specific scores. Estimated internal consistency of domain-specific scores ranged from *α* = 0.86 to 0.95 (Weziak-Bialowolska et al., 2021; Chen et al., 2022).

### 2.3 Outcomes (T2)

#### 2.3.1 Work distraction

A single item was used to assess distraction at work (Bialowolski et al., 2020). Participants used a five-point response scale (0%, 5–10%, 10–25%, 25–50%, 50–100%) to answer the question, “Thinking about your last week of work, what percent of the time did you feel distracted or not as productive as you would like?” Following previous studies (Bialowolski et al., 2020), we took the mid-point of the categories as the response value (i.e., 0, 7.5, 17.5, 37.5, 75%). Responses to this item were treated as a continuous score.

#### 2.3.2 Job satisfaction

A single item (i.e., “How satisfied are you with your job?”) was used to assess job satisfaction (Wanous et al., 1997). Participants responded to the item using an 11-point response scale (0 = *Least satisfied*; 10 = *Most satisfied*). We treated responses to this item as a continuous score.

### 2.4 Covariates (T1)

Following the modified disjunctive cause criterion for confounding to adjust for variables that might reasonably be a cause (or close proxy for a cause) of the predictor, the outcome, or both (VanderWeele, 2019), we adjusted for a range of covariates: demographic factors (age [≤ 30 years, 31–40 years, 41–50 years, and > 50 years], gender [female, male], race/ethnicity [non-Hispanic white, black/African American, other], marital status [single, married, divorced, widowed, separated, non-married partner]), socioeconomic status (educational attainment [high school, some college, associated degree, bachelor degree, graduate degree], house ownership [yes, no], midpoint annual salary bands [data obtained from the personnel files provided by human resource department of the employer]), number of health conditions (continuous variable; data obtained from the health care insurance files), family caregiving responsibilities (number of children under the age of 18 years [0, 1, 2, 3, 4, ≥ 5], caregiving to older persons at home [yes, no]), work-related characteristics (work hours per day [< 8 h, 8, 9–10, 11–12, 13–14, > 14], work from home [0 days/week, 1–4, 5], work type [exempt, non-exempt], meaning in work [continuous variable; range 0–10], workplace recognition [continuous variable; range 0–10], workplace supportive relationships [continuous variable; range 0–10], organizational productivity/work engagement [continuous variable; range 0–10]), religion/spirituality (religious service attendance [never, once every few months or once a year, 1–3 times a month, once a week, daily or more than once a week], spiritual practices [never, once every few months or once a year, 1–3 times a month, once a week, not daily but more than once a week, daily]), and civic engagement (participation in community groups [never, once every few months or once a year, 1–3 times a month, once a week, daily or more than once a week], volunteering [never, once every few months or once a year, 1–3 times a month, once a week, daily or more than once a week], voted in the last presidential election [yes, no, not a registered voter]). To explain more concretely why we included these covariates in the regression models, we present age as an example. Age is a potential confounder for the association in question because prior research has shown that it is associated with both the predictor of well-being (Shiba et al., 2022) and the outcomes of work distraction (Chen et al., 2023) and job satisfaction (Dobrow et al., 2018). To reduce the risk of reverse causation, we also adjusted for baseline values of both outcomes (VanderWeele et al., 2020).

### 2.5 Statistical processing

All statistical analyses were performed in SAS 9.4. In our primary analysis, we fit a series of lagged linear regressions estimating the association of each well-being domain at T1 with each work outcome at T2 (one well-being domain and one work outcome at a time). There were 217 participants with missing data on the predictors or covariates; missing data were imputed using multiple imputation via chained equations (*m* = 5). We adjusted for the full set of T1 covariates and T1 values of both work outcomes. For ease of interpretation, we standardized all continuous variables (mean = 0, standard deviation = 1) and reported standardized betas. Our descriptions of effect sizes are guided by benchmarks provided by Funder and Ozer (2019). Our inspection of the data suggested that modeling assumptions of linearity, normality, homoscedasticity, and non-collinearity were appropriately met. Unless otherwise specified, we focus our interpretation on the results of the primary analysis.

We conducted three sensitivity analyses, two of which applied more conservative approaches to covariate adjustment. First, we replicated each regression model in the primary analysis while further adjusting for a composite index comprising the average of the other five domain-specific well-being scores at T1 (i.e., those domains that were not entered as the predictor in a given model). Second, we repeated the primary analysis while including all six domain-specific well-being scores at T1, simultaneously. In the third analysis, we replicated the primary analysis using complete cases.

## 3 Results

Sample characteristics at baseline for this brief report are reported in Table 1. Participants were mostly 31 to 50 years old (58.6%), female (84.6%), white (74.0%), married or in a partnership (71.0%), and owned a home (72.2%). The midpoint salary band was $73,044.39 (*SD* = $34,129.38). Almost all participants worked at least 8 h per day (< 8 h: 1.2%), and a plurality worked from home 5 days per week (42.8%).

**Table 1:** Participant characteristics at T1 (*N* = 1,234).

Participant characteristics	*M* (SD) or %
Well-being	
Emotional health (range: 0–10)	7.57 (1.56)
Physical health (range: 0–10)	7.82 (1.67)
Meaning & purpose (range: 0–10)	7.98 (1.53)
Character strengths (range: 0–10)	7.93 (1.17)
Social connectedness (range: 0–10)	7.37 (1.74)
Financial security (range: 0–10)	6.32 (2.64)
Work distraction (range: 0–0.75)	0.11 (0.12)
Job satisfaction (range: 0–10)	7.30 (2.05)
Age categories, %	
30 years or below	11.83
31–50 years	58.59
50+ years	29.58
Female, %	84.60
Race/ethnicity, %	
Non-Hispanic White	74.07
Black/African American	12.24
Other	13.70
Married or in partnership, %	70.97
Educational attainment, %	
High school diploma or equivalent	7.87
Some college but not degree	22.95
College degree	48.61
Graduate degree	20.57
Number of children under the age of 18 years, %	
0	52.05
1	20.94
2	17.98
3	6.49
4	1.81
5+	0.74
Caregiving to older persons at home, %	27.35
House ownership, %	72.19
Mid-point salary bands ($) (range: $33,787.81–$246,979.16)^δ^	73,044.39 (34,129.38)
Number of health conditions (range: 0–12)^±^	1.88 (2.29)
Religious service attendance, %	
Never	27.93
Less than once per week	51.43
At least once per week	20.64
Spiritual practices, %	
Never	8.27
Less than once per week	30.61
At least once per week	61.13
Participation in community groups, %	
Never	31.80
Less than once per week	49.67
At least once per week	18.52
Volunteering, %	
Never	26.93
Less than once per week	63.55
At least once per week	9.52
Voted in the last presidential election, %	
Yes	82.25
No	14.13
Not a registered voter	3.62
Work hours (hours/day), %	
< 8 h	1.23
8 h	50.86
9–10 h	35.63
>10 h	12.29
Work from home (days/week), %	
0 day	30.48
1 to 4 days	26.71
5 days	42.81
Work type (exempt), %	63.53
Meaning in work (range: 0–10)	7.55 (2.10)
Workplace recognition (range: 0–10)	6.99 (2.59)
Workplace supportive relationships (range: 0–10)	7.63 (2.41)
Organizational productivity/work engagement (range: 0–10)	7.35 (1.86)

M, mean; SD, standard deviation. ^δ^Data on mid-point salary bands were obtained from the human resource department of the employer. ^±^Data on the number of health conditions were obtained from medical claims.

Pearson correlations among the primary study variables are reported in Supplementary Table S2. The domains of well-being evidenced small-to large-sized cross-sectional correlations with lower work distraction (*r*s = −0.31 to −0.15, *p*s < 0.001) and higher job satisfaction (*r*s = 0.18 to 0.41, *p*s < 0.001) at T1. Prospective correlations between domains of well-being at T1 and the outcomes at T2 were slightly smaller (work distraction: *r*s = −0.26 to −0.11, *p*s < 0.001; job satisfaction: *r*s = 0.14 to 0.31, *p*s < 0.001).

Results of the primary analysis are reported in Table 2. There was evidence of a small negative association between emotional health and subsequent work distraction (*t* = −3.89, *β* = −0.11, 95% CI = −0.17, −0.06, *p* < 0.001), with slightly smaller negative associations found for meaning & purpose (*t* = −2.66, *β* = −0.08, 95% CI = −0.14, −0.02, *p* = 0.008), social connectedness (*t* = −2.64, *β* = −0.08, 95% CI = −0.13, −0.02, *p* = 0.008), and character strengths (*t* = −2.40, *β* = −0.07, 95% CI = −0.12, −0.01, *p* = 0.016). Associations of physical health (*t* = −1.47, *β* = −0.04, 95% CI = −0.10, 0.01, *p* = 0.143) and financial security (*t* = −0.94, *β* = −0.03, 95% CI = −0.08, 0.03, *p* = 0.349) with subsequent work distraction data were negligible, and neither met the conventional *p* < 0.05 threshold for statistical significance.

**Table 2:** Longitudinal associations between well-being domains (T1) and subsequent work outcomes assessed approximately 1 year later (*N* = 1,234).

Well-being domains (T1)	Work outcomes (T2)	
	Work distraction^a^ *β* (95% CI)	Job satisfaction^b^ *β* (95% CI)
Emotional health	−0.11 (−0.17, −0.06)***	0.08 (0.03, 0.13)**
Physical health	−0.04 (−0.10, 0.01)	0.02 (−0.04, 0.07)
Meaning & purpose	−0.08 (−0.14, −0.02)**	0.05 (−0.00, 0.11)
Character strengths	−0.07 (−0.12, −0.01)*	0.02 (−0.03, 0.07)
Social connectedness	−0.08 (−0.13, −0.02)**	0.08 (0.03, 0.13)**
Financial security	−0.03 (−0.08, 0.03)	0.05 (0.00, 0.11)*

A set of separate linear regression models were used to regress each of the dependent variables at T2 on the domain-specific well-being scores at T1 (one well-being domain and one outcome at a time). All models adjusted for age, gender, race/ethnicity, marital status, socioeconomic status (educational attainment, house ownership, midpoint annual salary bands), number of health conditions, family caregiving responsibilities (number of children under the age of 18 years, caregiving to older persons at home), work-related characteristics (work hours per day, work from home, work type, meaning in work, workplace recognition, workplace supportive relationships, organizational productivity/work engagement), religion/spirituality (religious service attendance, spiritual practices), and civic engagement (participation in community groups, volunteering, voted in the last presidential election) assessed at T1, as well as T1 values of both work outcomes. ^a^Model fit statistics for the six models were *F*s = 11.18 to 11.66, *p*s < 0.001, *R*^2^ = 0.278 to 0.286. ^b^Model fit statistics for the six models were *F*s = 16.68 to 17.03, *p*s < 0.001, *R*^2^ = 0.365 to 0.369. *p < 0.05, **p < 0.01, ***p < 0.001.

Emotional health (*t* = 2.96, *β* = 0.08, 95% CI = 0.03, 0.13, *p* = 0.003) and social connectedness (*t* = 3.10, *β* = 0.08, 95% CI = 0.03, 0.13, *p* = 0.002) evidenced small positive associations with subsequent job satisfaction. Slightly smaller positive associations were found for financial security (*t* = 1.98, *β* = 0.05, 95% CI = 0.00, 0.11, *p* = 0.048) and meaning & purpose (*t* = 1.92, *β* = 0.05, 95% CI = −0.00, 0.11, *p* = 0.055), although the latter did not meet the conventional *p* < 0.05 threshold for statistical significance. Associations of physical health (*t* = 0.60, *β* = 0.02, 95% CI = −0.04, 0.07, *p* = 0.549) and character strengths (*t* = 0.70, *β* = 0.02, 95% CI = −0.03, 0.07, *p* = 0.485) with subsequent job satisfaction were negligible, and neither met the conventional *p* < 0.05 threshold for statistical significance.

In the sensitivity analyses that also adjusted for a composite well-being score comprising the five other domains that were not examined as the predictor variable (see Supplementary Table S3), effect sizes for the associations of emotional health with subsequent work distraction, emotional health with subsequent job satisfaction, and social connectedness with subsequent job satisfaction remained comparable to the results of the primary analysis; effect sizes for other associations attenuated more substantially. A similar pattern of findings emerged when analyses additionally adjusted for all domains of well-being simultaneously (see Supplementary Table S4). Results of the complete-case analysis were largely comparable to the results of the primary analysis (see Supplementary Table S5).

## 4 Discussion

Results of the primary analysis in this brief research report showed that several domains of well-being were associated prospectively with both lower work distraction and higher job satisfaction, but associations with each outcome did vary to some extent by domain. Taken together, the findings of this study build on prior research that has typically focused on a single or few indicators of well-being as predictors of work outcomes.

We found that one more domain of well-being showed evidence of an association with work distraction than with job satisfaction (4 vs. 3 associations in the primary analysis that passed the *p* < 0.05 threshold), although effect sizes for the associations were generally small. Emotional health and social connectedness were the strongest predictors of both work distraction and job satisfaction. Our findings resonate with prior cross-sectional (e.g., Staw et al., 1994; Spector, 1997; Cote, 1999; Russell, 2008) and longitudinal studies (e.g., Staw et al., 1994; Unanue et al., 2017; Bialowolski and Weziak-Bialowolska, 2021) that have reported evidence showing that indicators of emotional health (e.g., life satisfaction, positive affect) are related to better work outcomes (e.g., work performance, cooperation with other employees). Similarly, prior cross-sectional work has demonstrated the importance of social interaction in life (e.g., church attendance, volunteer work, meeting with friends) for job satisfaction (Fiorillo and Nappo, 2014). Although there are theoretical and empirical studies that suggest that social interaction at work is related to productivity (Turner et al., 2002; Russell, 2008; Cornelissen, 2016), our study is one of the first to provide evidence supporting a longitudinal association between social connectedness in life and higher subsequent job satisfaction.

There was some variation in the pattern of associations that were observed across the outcomes. For example, in the primary analysis, character strengths and meaning & purpose were associated with lower subsequent work distraction (but not job satisfaction), whereas financial security was associated with lower subsequent job satisfaction (but not work distraction). This pattern of findings suggests that different domains of well-being might be more strongly related to some employee work outcomes than others, a finding that could be of practical significance to organizations that are interested in identifying opportunities to improve employee productivity and satisfaction with their work. For example, tailored interventions that target specific domains of employee well-being may be needed to achieve desired effects on particular work outcomes.

This study’s findings point to some potential implications for employees that could be addressed at the organizational level, given that decisions about the work environment can improve employee satisfaction, productivity, and well-being (Danielsson and Bodin, 2008; van der Voordt and Jensen, 2021). Because different domains of well-being can affect work in unique ways, it may be important for organizational leadership to consider well-being as a multidimensional concept because it can inform decisions about targeted initiatives that may be particularly useful for supporting employees. In organizational settings where resources are more limited, it may be prudent for leadership to prioritize workplace initiatives that aim to improve employees’ emotional health and social connectedness. Although the effect sizes that we observed for these domains of well-being were small, organizational leadership might consider these directions because even small effect sizes can translate to large population impact under certain conditions (Matthay et al., 2021; Komura et al., 2023). For example, if interventions to promote emotional health or social connectedness are disseminated throughout a large organization, the implications for reducing work distraction and increasing job satisfaction within the organization could be quite substantial. One potential avenue that organizations could pursue to support the emotional health of their employees is to foster a psychological climate of caring (Cowden et al., 2022), which includes perceptions of collective pattern of fair treatment and recognition, trustworthy leadership, and respect (Weziak-Bialowolska et al., 2023). Organizations might also consider scheduling events and offering opportunities before, during, or after regular working hours to promote emotional health and build a sense of community in the workplace (Cornelissen, 2016; Latino et al., 2021). Furthermore, flexible work schedules and arrangements, such as opportunities to work from home, might improve emotional health and social connectedness by providing more opportunities for employees to spend more time with their families (Chen et al., 2023). Whether such strategies lead to practically significant changes in the work distraction and job satisfaction of employees within a particular organization might depend on several factors, such as existing levels of employee well-being and the size of the organization.

### 4.1 Strengths, limitations, and future research directions

A key strength of the present study is the prospective study design in which there was a clear temporal order between predictors and outcomes; under certain assumptions, the two-wave longitudinal design employed in this study allows for estimation of potential causal relationships between well-being and work outcomes (VanderWeele et al., 2020). Our adjustment for baseline work outcomes at T1 also mitigated concerns of reverse causation. However, there are selected limitations that warrant consideration. First, the sample included employees working at a large, national health insurance company. Given that the data used in the present study are not nationally representative, our findings might be more generalizable to certain types of organizations (e.g., national home and auto insurance companies) and employment roles (e.g., desk jobs) compared to others. Furthermore, our sample was predominantly female, non-Hispanic White, and in middle adulthood (31–50 years). Replication studies are needed to evaluate whether our findings generalize to specific subpopulations (e.g., males, racial/ethnic minorities) and are transferable to other workplace settings, geographic contexts, and socioeconomic conditions.

Second, with observational data, there is a possibility that the results may be biased by unmeasured confounding (e.g., perceived organizational fit). We mitigated this by adjusting for an array of potential confounders, including those that might be missing from other studies and those that might act as proxies for unmeasured confounders. Relatedly, approximately 40% of participants were lost to follow-up. If retained participants differed systematically from those who dropped out of the study, this attrition can lead to selection bias. Unknown extraneous variables (e.g., negative life events) may have impacted non-response to follow-up as well as responses to the follow-up survey among those who were retained. Hence, measured or unmeasured factors could have biased the results and weakened the generalizability of our findings.

Third, the findings of this study should be interpreted in light of the one-year time lag that was employed. A shorter or longer lag may be needed to identify the maximum lagged effect of well-being on the work outcomes that we examined (Dormann and Griffin, 2015). Future studies might consider supplementing our findings with longitudinal designs that consider the optimal time lag that might be needed to detect maximum effect sizes.

Fourth, we relied on self-report data to measure predictors, outcomes, and most covariates, which may be subject to response bias. Some of these concerns are ameliorated by employing a well-validated measure of multidimensional well-being. However, future research might consider expanding on our brief assessment of job satisfaction by measuring specific dimensions of the construct (e.g., satisfaction with managers, colleagues, work environment) and comparing self-reported outcomes to more objective markers of employee performance (e.g., achievement of key performance indicators).

Fifth, each of the work outcomes was assessed using a single item, and neither explain how and why people are distracted at work or satisfied with their job. The work distraction item had a response scale with two options that overlapped slightly (e.g., 5–10% vs. 10–25%), which may have introduced some inconsistencies in the way participants responded to the item. However, the purpose of the response scale was not to obtain an exact proportion of work distraction but rather to assess a general level of work distraction that an employee perceived. To mitigate potential concerns about the response scale for this item, we leveraged the midpoint of each percentage range. Future studies might consider using a different set of response categories.

Sixth, the results for the work distraction outcome should be considered in light of the item that was used, which also captures perceived unproductivity. Given the complicated relationship between work distraction and its immediate causes revealed in prior studies (e.g., Wajcman and Rose, 2011; Yin et al., 2018; Bialowolski et al., 2020; Orhan et al., 2021), additional research is needed to explore the boundary conditions (moderators) and mechanisms (mediators) that might improve our understanding of the relationship between well-being and work distraction. Another possible avenue for future exploration is whether and how the individual domains of well-being influence each other to shape work outcomes (Ohrnberger et al., 2017).

## 5 Conclusion

The present brief report provides some evidence indicating that several domains of well-being are related to improved subsequent work distraction and job satisfaction. Although further work is needed to improve our understanding of linkages between multidimensional well-being and various work outcomes, our findings suggest that organizations might consider prioritizing opportunities to promote the emotional health and social connectedness of its employees.

## Data availability statement

The datasets presented in this study can be found in online repositories. The names of the repository/repositories and accession number(s) can be found at: Harvard Dataverse repository: https://doi.org/10.7910/DVN/ZJEEN5.

## Ethics statement

The studies involving humans were approved by Harvard T.H. Chan School of Public Health Institutional Review Board. The studies were conducted in accordance with the local legislation and institutional requirements. The participants provided their written informed consent to participate in this study.

## Author contributions

EF: Conceptualization, Writing – original draft, Writing – review & editing. RC: Conceptualization, Writing – original draft, Writing – review & editing. YC: Formal analysis, Writing – review & editing. DW-B: Data curation, Writing – review & editing. PB: Data curation, Writing – review & editing. ML: Data curation, Writing – review & editing. EM: Conceptualization, Data curation, Funding acquisition, Investigation, Methodology, Writing – review & editing. TV: Conceptualization, Data curation, Funding acquisition, Investigation, Methodology, Writing – review & editing.
