# Supplementary material for: Prospective associations of multidimensional well-being with work distraction and job satisfaction: a two-wave study of US employees
Source: Front Psychol. 2024 Jan 26;15:1326655. doi: 10.3389/fpsyg.2024.1326655 (PMC10853318; doi:10.3389/fpsyg.2024.1326655)
Supplement: Supplementary file 1 [file Table_1.DOCX]

Prospective Associations of Multidimensional Well-Being with Work Distraction and Job Satisfaction: A Two-Wave Study of US Employees

**SUPPLEMENTARY MATERIAL**

| Supplemental Table S1  *Six Well-Being Assessment Components and 40 Items* | | |
| --- | --- | --- |
| Domain | Code | Question and response scale |
| Emotional Health | EH1 | Overall, how satisfied are you with life as a whole these days?  (0 = Not Satisfied at All, 10 = Completely Satisfied) |
| Emotional Health | EH2 | On average, how happy have you felt during the last 7 days?  (0 = Extremely Unhappy, 10 = Extremely Happy) |
| Emotional Health | EH3 | I expect more good things in my life than bad.  (0=Strongly Disagree, 10=Strongly Agree) |
| Emotional Health | EH4 | How would you rate your overall mental health? (0 = Poor, 10 = Excellent) |
| Emotional Health | EH5 | Are you depressed? (0=Not at all Depressed, 10=Very Depressed) (r) |
| Emotional Health | EH6 | Do you have anxiety that keeps you from doing the things in life that you need to do? (0=Never, 10=Always) (r) |
| Emotional Health | EH7 | In stressful situations, I manage my emotions so that I am still in control of myself. (0=Strongly Disagree, 10=Strongly Agree) |
| Physical Health | PH1 | In general, how would you rate your physical health?  (0 = Poor, 10 = Excellent) |
| Physical Health | PH2 | I have no major illnesses or injuries.  (0=Strongly Disagree, 10=Strongly Agree) |
| Physical Health | PH3 | I do not routinely get sick. (0=Strongly Disagree, 10=Strongly Agree) |
| Physical Health | PH4 | My health does not prevent me from doing what I would like.  (0=Strongly Disagree, 10=Strongly Agree) |
| Physical Health | PH5 | My pain makes it hard for me to do my usual activities.  (0=Strongly Disagree, 10=Strongly Agree) (r) |
| Physical Health | PH6 | Based on my past health, I expect to be healthy long into the future. (0=Strongly Disagree, 10=Strongly Agree) |
| Physical Health | PH7 | I regularly do things to maintain and improve my health, in diet, exercise, and health care (0=Strongly Disagree, 10=Strongly Agree) |
| Meaning and Purpose | MP1 | I know what gives meaning to my life.  (0=Strongly Disagree, 10=Strongly Agree) |
| Meaning and Purpose | MP2 | I have values and beliefs that help me understand who I am.  (0=Strongly Disagree, 10=Strongly Agree) |
| Meaning and Purpose | MP3 | My life has a clear sense of purpose.  (0=Strongly Disagree, 10=Strongly Agree) |
| Meaning and Purpose | MP4 | I understand my purpose in life. (0=Strongly Disagree, 10=Strongly Agree) |
| Meaning and Purpose | MP5 | Overall, to what extent do you feel the things you do in your life are worthwhile? (0 = Not at All Worthwhile, 10 = Completely Worthwhile) |
| Meaning and Purpose | MP6 | I am pursuing what is most important to me in my life.  (0=Strongly Disagree, 10=Strongly Agree) |
| Character Strengths | CS1 | I always act to promote good in all circumstances, even in difficult and challenging situations. (0=Not True of Me; 10=Completely True of Me) |
| Character Strengths | CS2 | I always know the right thing to do.  (0=Not True of Me; 10=Completely True of Me) |
| Character Strengths | CS3 | I always treat everyone with kindness, fairness and respect.  (0=Not True of Me; 10=Completely True of Me) |
| Character Strengths | CS4 | I am always able to give up some happiness now for greater happiness later. (0=Not True of Me; 10=Completely True of Me) |
| Character Strengths | CS5 | I am willing to face difficulties in order to do what is right.  (0=Not True of Me; 10=Completely True of Me) |
| Character Strengths | CS6 | I give up personal pleasures whenever it is possible to do some good instead. (0=Not True of Me; 10=Completely True of Me) |
| Character Strengths | CS7 | I get to use my strengths to help others.  (0=Not True of Me; 10=Completely True of Me) |
| Social Connectedness | SC1 | My relationships are as satisfying as I would want them to be.  (0=Strongly Disagree, 10=Strongly Agree) |
| Social Connectedness | SC2 | There are people who really understand me. (0=Never, 10=Often) |
| Social Connectedness | SC3 | How often do you feel lonely? (0=Never, 10=Almost Always) (r) |
| Social Connectedness | SC4 | I am content with my friendships and relationships.  (0=Strongly Disagree, 10=Strongly Agree) |
| Social Connectedness | SC5 | I have enough people I feel comfortable asking for help at any time. (0=Strongly Disagree, 10=Strongly Agree) |
| Social Connectedness | SC6 | I feel connected to the broader community around me.  (0=Strongly Disagree, 10=Strongly Agree) |
| Social Connectedness | SC7 | People in my broader community trust and respect one another. (0=Strongly Disagree, 10=Strongly Agree) |
| Financial Security | FS1 | I am able to meet my normal monthly living expenses without any difficulty. (0=Completely Disagree, 10=Completely Agree) |
| Financial Security | FS2 | How often do you worry about food, housing, or health expenses? (0=Do Not Ever Worry, 10=Worry All of the Time) (r) |
| Financial Security | FS3 | I have sufficient savings that I could cover six months of expenses. (0=Strongly Disagree, 10=Strongly Agree) |
| Financial Security | FS4 | My financial circumstances give me freedom to pursue my goals. (0=Strongly Disagree, 10=Strongly Agree) |
| Financial Security | FS5 | Given my age, I have done adequate financial planning for the future. (0=Strongly Disagree, 10=Strongly Agree) |
| Financial Security | FS6 | The amount of debt I have often overwhelms me. (0=Strongly Disagree, 10=Strongly Agree) (r) |
| *Note*. (r) reverse-coded item. | | |

| Supplemental Table S2  *Pearson Correlations Among Primary Study Variables* | | | | | | | | | |
| --- | --- | --- | --- | --- | --- | --- | --- | --- | --- |
| Variable | (1) | (2) | (3) | (4) | (5) | (6) | (7) | (8) | (9) |
| T1 |  |  |  |  |  |  |  |  |  |
| (1) Emotional health |  |  |  |  |  |  |  |  |  |
| (2) Physical health | 0.54 |  |  |  |  |  |  |  |  |
| (3) Meaning & purpose | 0.72 | 0.44 |  |  |  |  |  |  |  |
| (4) Character strengths | 0.52 | 0.35 | 0.64 |  |  |  |  |  |  |
| (5) Social connectedness | 0.67 | 0.45 | 0.71 | 0.58 |  |  |  |  |  |
| (6) Financial security | 0.46 | 0.44 | 0.36 | 0.23 | 0.40 |  |  |  |  |
| (7) Work distraction | -0.31 | -0.17 | -0.25 | -0.22 | -0.25 | -0.15 |  |  |  |
| (8) Job satisfaction | 0.35 | 0.18 | 0.41 | 0.29 | 0.36 | 0.22 | -0.29 |  |  |
| T2 |  |  |  |  |  |  |  |  |  |
| (9) Work distraction | -0.26 | -0.12 | -0.22 | -0.19 | -0.21 | -0.11 | 0.46 | -0.23 |  |
| (10) Job satisfaction | 0.29 | 0.14 | 0.30 | 0.22 | 0.31 | 0.18 | -0.23 | 0.56 | -0.32 |
| *Note*. All correlations statistically significant at *p* < .001. | | | | | | | | | |

| Supplemental Table S3  *Longitudinal Associations Between Well-being Domains (T1) and Subsequent Work Outcomes Assessed Approximately One Year Later (Additionally Adjusting for the Composite Score of all Other Domains;* N *= 1,234)* | | |
| --- | --- | --- |
| Well-being domains (T1) | Work outcomes (T2) | |
|  | Work distraction  β (95% CI) | Job satisfaction  β (95% CI) |
| Emotional health | -0.11 (-0.19, -0.04)** | 0.07 (-0.01, 0.14) |
| Physical health | 0.00 (-0.06, 0.07) | -0.03 (-0.09, 0.03) |
| Meaning and purpose | -0.04 (-0.12, 0.04) | 0.01 (-0.07, 0.08) |
| Character strengths | -0.04 (-0.10, 0.03) | -0.03 (-0.09, 0.03) |
| Social connectedness | -0.04 (-0.11, 0.04) | 0.07 (0.00, 0.14)* |
| Financial security | 0.02 (-0.05, 0.08) | 0.03 (-0.03, 0.09) |
| *Note*. A set of separate linear regression models were used to regress each of the dependent variables at T2 on the domain-specific well-being scores at T1 (one well-being domain and one outcome at a time). All models adjusted for age, gender, race/ethnicity, marital status, socioeconomic status (educational attainment, house ownership, midpoint annual salary bands), number of health conditions, family caregiving responsibilities (number of children under the age of 18 years, caregiving to older persons at home), work-related characteristics (work hours per day, work from home, work type, meaning in work, workplace recognition, workplace supportive relationships, organizational productivity/work engagement), religion/spirituality (religious service attendance, spiritual practices), and civic engagement (participation in community groups, volunteering, voted in the last presidential election) assessed at T1, a composite score comprising other well-being domains (that were not examined as the exposure variable in each model) assessed at T1, as well as T1 values of both work outcomes. **p* < .05, ***p* < .01. | | |

| Supplemental Table S4  *Longitudinal Associations Between Well-being Domains (T1) and Subsequent Work Outcomes Assessed Approximately One Year Later (Additionally Adjusting for All Other Domains of Well-being;* N *= 1,234)* | | |
| --- | --- | --- |
| Well-being domains (T1) | Work outcomes (T2) | |
|  | Work distraction  β (95% CI) | Job satisfaction  β (95% CI) |
| Emotional health | -0.11 (-0.19, -0.03)** | 0.07 (-0.01, 0.15) |
| Physical health | 0.01 (-0.05, 0.08) | -0.04 (-0.10, 0.02) |
| Meaning and purpose | 0.01 (-0.08, 0.10) | -0.02 (-0.10, 0.06) |
| Character strengths | -0.03 (-0.10, 0.04) | -0.03 (-0.10, 0.03) |
| Social connectedness | -0.01 (-0.09, 0.07) | 0.07 (-0.00, 0.15) |
| Financial security | 0.02 (-0.05, 0.08) | 0.03 (-0.03, 0.09) |
| *Note*. Separate linear regression models were used to regress each of the dependent variables at T2 on all domain-specific well-being scores at T1 (one outcome at a time). All models adjusted for age, gender, race/ethnicity, marital status, socioeconomic status (educational attainment, house ownership, midpoint annual salary bands), number of health conditions, family caregiving responsibilities (number of children under the age of 18 years, caregiving to older persons at home), work-related characteristics (work hours per day, work from home, work type, meaning in work, workplace recognition, workplace supportive relationships, organizational productivity/work engagement), religion/spirituality (religious service attendance, spiritual practices), and civic engagement (participation in community groups, volunteering, voted in the last presidential election) assessed at T1, as well as T1 values of both work outcomes. ***p* < .01. | | |

| Supplemental Table S5  *Longitudinal Associations Between Well-being Domains (T1) and Subsequent Work Outcomes Assessed Approximately One Year Later (Using Complete Cases;* N *= 1,014)* | | |
| --- | --- | --- |
| Well-being domains (T1) | Work outcomes (T2) | |
|  | Work distraction  β (95% CI) | Job satisfaction  β (95% CI) |
| Emotional health | -0.11 (-0.17, -0.05)*** | 0.07 (0.02, 0.13)* |
| Physical health | -0.04 (-0.10, 0.03) | 0.01 (-0.05, 0.06) |
| Meaning and purpose | -0.09 (-0.16, -0.03)** | 0.05 (-0.01, 0.11) |
| Character strengths | -0.07 (-0.13, -0.00)* | 0.02 (-0.03, 0.08) |
| Social connectedness | -0.08 (-0.14, -0.02)* | 0.09 (0.03, 0.15)** |
| Financial security | -0.04 (-0.10, 0.03) | 0.05 (-0.01, 0.11) |
| *Note*. A set of separate linear regression models were used to regress each of the dependent variables at T2 on the domain-specific well-being scores at T1 (one well-being domain and one outcome at a time). All models adjusted for age, gender, race/ethnicity, marital status, socioeconomic status (educational attainment, house ownership, midpoint annual salary bands), number of health conditions, family caregiving responsibilities (number of children under the age of 18 years, caregiving to older persons at home), work-related characteristics (work hours per day, work from home, work type, meaning in work, workplace recognition, workplace supportive relationships, organizational productivity/work engagement), religion/spirituality (religious service attendance, spiritual practices), and civic engagement (participation in community groups, volunteering, voted in the last presidential election) assessed at T1, as well as T1 values of both work outcomes.  **p* < .05, ***p* < .01, ****p* < .001. | | |
